# Establishment and Verification of a Perioperative Blood Transfusion Model After Posterior Lumbar Interbody Fusion: A Retrospective Study Based on Data From a Local Hospital

**DOI:** 10.3389/fsurg.2021.695274

**Published:** 2021-08-30

**Authors:** Bo Liu, Junpeng Pan, Hui Zong, Zhijie Wang

**Affiliations:** ^1^Department of Spinal Surgery, The Affiliated Hospital of Qingdao University, Qingdao, China; ^2^Department of Neurology, The People's Hospital of Qingyun, Dezhou, China

**Keywords:** posterior lumbar interbody fusion, blood transfusion, risk factors, nomogram, a retrospective study

## Abstract

**Objective:** We aimed to analyze the related risk factors for blood transfusion and establish a blood transfusion risk model during the per-ioperative period of posterior lumbar interbody fusion (PLIF). It could provide a reference for clinical prevention and reduction of the risk of blood transfusion during the peri-operative period.

**Methods:** We retrospectively analyzed 4,378 patients who underwent PLIF in our hospital. According to whether they were transfused blood or not, patients were divided into the non-blood transfusion group and the blood transfusion group. We collected variables of each patient, including age, sex, BMI, current medical history, past medical history, surgical indications, surgical information, and preoperative routine blood testing. We randomly divide the whole population into training group and test group according to the ratio of 4:1. We used the multivariate regression analyses get the independent predictors in the training set. The nomogram was established based on these independent predictors. Then, we used the AUC, calibration curve and DCA to evaluate the nomogram. Finally, we verified the performance of the nomogram in the validation set.

**Results:** Three or more lumbar fusion segments, preoperative low hemoglobin, with hypertension, lower BMI, and elder people were risk factors for blood transfusion. For the training and validation sets, the AUCs of the nomogram were 0.881 (95% CI: 0.865–0.903) and 0.890 (95% CI: 0.773–0.905), respectively. The calibration curve shows that the nomogram is highly consistent with the actual observed results. The DCA shows that the nomogram has good clinical application value. The AUC of the nomogram is significantly larger than the AUCs of independent risk factors in the training and validation set.

**Conclusion:** Three or more lumbar fusion segments, preoperative low hemoglobin, with hypertension, lower BMI, and elder people are associated with blood transfusion during the peri-operative period. Based on these factors, we established a blood transfusion nomogram and verified that it can be used to assess the risk of blood transfusion after PLIF. It could help clinicians to make clinical decisions and reduce the incidence of peri-operative blood transfusion.

## Introduction

Posterior lumbar interbody fusion (PLIF) is a classic surgical method currently used by clinicians to treat lumbar degenerative diseases such as lumbar disc herniation, lumbar spinal stenosis, and lumbar spondylolisthesis (1). PLIF was reported to have a significant effect on relieving lumbar pain and improving radicular symptoms of the lower extremities. It can achieve good segmental fusion and fixation. In addition, it can completely restore the height of the intervertebral space to maintain the physiological curvature of the spine (2–4). However, PLIF is an open operation, during which the muscles on both sides of the spinous process need to be completely stripped. There are many blood vessels running in this area, and some areas of the blood vessels will inevitably be damaged during the operation (5).

The total blood loss during the peri-operative period is approximately 800–1,000 ml, and with the increase in the surgical segment, the blood loss during the peri-operative period also increases (6, 7). In clinical practice, the traditional method to solve severe per-ioperative anemia is allogeneic blood transfusion, which can quickly alleviate the condition and correct anemia (8, 9). However, it may bring about a series of problems, such as increased economic burden, iatrogenic infection, and postoperative complication rate (10). Especially for middle-aged and elderly patients during the peri-operative period, massive bleeding affects the heart, kidney, lung and other functions, leading to abnormal blood coagulation, incision infection, organ insufficiency and a series of complications (11, 12).

At present, nomograms are widely used in the prognosis and diagnosis of clinical medicine. In a nomogram, multiple risk factors can be combined to predict the probability of an outcome, and the results can be visualized. Nomogram is widely used in the diagnosis and prognosis of diseases. It can integrate multiple risk factors to make a comprehensive assessment of the risk of diseases, and visualize the results to make them easy to understand (13). After consulting the literature, we found that there are relatively few studies on the risk factors for blood transfusion during the peri-operative period of PLIF, and no researchers have established and verified the clinical prediction model of blood transfusion after PLIF. Therefore, this study aims to investigate the risk factors for postoperative blood transfusion and the incidence of blood transfusion in patients with PLIF for the treatment of lumbar degenerative diseases and to establish and verify a predictive nomogram of postoperative blood transfusion on this basis.

## Methods

### Collection of Patients' Clinical Data

We retrospectively analyzed the clinical diagnosis and treatment data of patients who underwent PLIF. All of the patients underwent a standard posterior spinal fusion. This study was approved by the Ethics Committee of the Affiliated Hospital of Qingdao University. From January 2015 to December 2020, 5069 patients were in compliance with the requirements. Of these, 691 were excluded: 319 patients with incomplete clinical data such as blood routine training results; 247 were second revision surgery and were diagnosised of lumbar infectious diseases; 68 patients with severe complications within 3 days after the operation, such as spinal cord injury, renal or liver dysfunction; 57 used anti-coagulant and anti-platelet drugs within 15 days. Ultimately, 4,378 patients were included in this study. Although clinical blood transfusion events are still controversial, the criteria of the blood transfusion group in our hospital is that hemoglobin was <70 g/L or hemoglobin was <80 g/L, but patients had symptoms of anemia within 14 days.

We collected variables of each patient, included demographic data, past medical history, concomitant diseases, surgical indications, preoperative routine blood examination, intraoperative fusion segments. The fusion segments were defined as the segments number of lumbar interbody fusion, it was only PLIF without decompression at other levels. All data were collected independently from the hospital's medical record system by two surgeons, and any disputed data were modified with the consent of the two physicians who extracted the data. The surgical method was standard PLIF, and the surgeons were all senior chief physicians in charge. Each patient entered the clinical path for unified process management. We randomly divide the whole population into training group and test group according to the ratio of 4:1.

### Statistical Analysis

All statistical analyses were performed in the R software (version 4.0.3, R Foundation for Statistical Computing, Austria). The normality of continuous variables was determined by Shapiro-Wilk training. Normally distributed data are represented by the Mean ± SD, non-normally distributed data are represented by the Quartiles and categorical data are represented by numbers or percentages. Univariate analysis was performed on the training set, the continuous variables were evaluated using Student's *t*-test or the Mann-Whitney *U*-test, while categorical variables were subjected to the chi-square test. Then, multivariate regression analysis was used on the training set to determine the independent predictors of blood transfusion after lumbar fusion. *P* < 0.05 (two-sided) was considered significant.

Meaningful variables of logistics multifactor regression analysis were included in R solfware, and a nomogram was constructed. The AUC of the ROC curve was used to illustrate the predictive ability of the model. The calibration curve is an image comparison between the predicted risk and the patient's true risk. The closer the predicted risk is to the standard curve, the better the compliance of the model. The decision curve analysis method was used to evaluate the net benefit and the effectiveness of the nomogram. Finally, in the training and validation sets, the independent nomogram and each meaningful variable subgroup were analyzed and compared, and the nomogram and the ROC curve of each independent predictor were generated to compare the predictive ability.

## Results

### Demographic Characteristics of the Patients

From January 2015 to December 2020, a total of 4,378 patients underwent lumbar fusion in the Affiliated Hospital of Qingdao University, of whom 256 patients had blood transfusions during the peri-operative period, and the blood transfusion rate during the perioperative period was 5.8%. Table 1 demonstrates the baseline characteristics. There was no significant difference between the two groups (*P* > 0.05). This shows that there is comparability between the two. Among them, there were 2,118 males and 2,260 females. The average age of the patients was 56.73 ± 14.11 years, and the average body mass index (BMI) was 25.64 ± 3.63 kg/m^2^. Among these patients, there were 1,731 patients with 1 fusion segment, 1,870 patients with two fusion segments, and 777 patients with three or more fusion segments.

**Table 1:** Comparison of demographic and preoperative data of the two groups of patients.

	**Non-transfusion (*n* = 4,122)**	**Transfusion (*n* = 256)**	***t*/*z*/χ^2^**	***p***
Sex			−0.986	0.324
Female	2,136 (51.8)	124 (48.4)		
Male	1,986 (48.2)	132 (51.6)		
Age (years)			135.697	<0.001
<55	1,658 (40.2)	24 (9.4)		
55–65	1,183 (28.7)	70 (27.3)		
>65	1,281 (31.1)	162 (63.3)		
BMI (kg/m^2^)	25.50 (23.30, 27.90)	24.20 (21.80, 26.72)	−5.995	<0.001
**Comorbidities (%)**				
Hypertension	1,392 (33.8)	110 (43.0)	−2.968	0.003
Diabetes mellitus	801 (19.4)	50 (19.5)	0.000	1.000
Coronary heart disease	571 (13.9)	43 (16.8)	−1.224	0.221
Cerebral thrombosis	33 (0.8)	2 (0.8)	0.000	1.000
Respiratory diseases	228 (5.5)	21 (8.2)	−1.650	0.099
Digestive system diseases	371 (9.0)	21 (8.2)	−0.321	0.748
Other	545 (13.2)	38 (14.8)	−0.646	0.518
Indications for surgery (%)			2.051	0.562
Lumbar disc herniation	1,776 (43.1)	115 (45.5)		
Spinal stenosis	1,170 (28.4)	71 (28.1)		
Lumbar spondylolisthesis	869 (21.1)	45 (17.8)		
Other	307 (7.4)	22 (8.6)		
**Previous history (%)**				
Surgical history	1,476 (35.8)	113 (44.1)	−2.612	0.009
Blood transfusion	106 (2.6)	4 (1.6)	−0.796	0.426
Allergies	80 (1.9)	3 (1.2)	−0.639	0.523
Smoking	681 (16.5)	37 (14.5)	−0.781	0.435
Alcohol	541 (13.1)	31 (12.1)	−0.372	0.710
ABO (%)			−1.296	0.195
A	1,206 (29.3)	62 (24.2)		
AB	446 (10.8)	32 (12.5)		
B	1,355 (32.9)	81 (31.6)		
O	1,115 (27.0)	81 (31.6)		
RH (%)			−0.451	0.652
Negative (–)	16 (0.4)	2 (0.8)		
Positive (+)	4,106 (99.6)	254 (99.2)		
**Laboratory tests**				
Hb	139.00 (129.00, 150.00)	128.00 (116.00, 138.00)	−11.166	<0.001
NRBC	5.02 (4.30, 5.65)	4.80 (4.17, 5.50)	−2.132	0.033
MCH	30.50 (29.50, 31.50)	30.60 (29.60, 31.83)	−2.108	0.035
MCHC	339.00 (332.00, 346.00)	339.00 (331.00, 346.25)	−0.020	0.984
MCV	89.80 (87.10, 92.40)	90.10 (87.60, 93.62)	−2.366	0.018
WBC	6.16 (5.16, 7.51)	5.94 (5.08, 6.98)	−2.308	0.021
PLT	131.00 (96.00, 171.00)	133.50 (97.75, 165.00)	−0.429	0.668
Decompression Fusion Segment			407.256	<0.001
1	1,695 (41.1)	36 (14.1)		
2	1,815 (44.0)	55 (21.5)		
≥3	612 (14.8)	165 (64.5)		

### Independent Risk Factors for Blood Transfusion in Training Set

In the training set, 205 patients received blood transfusion within 14 days after lumbar fusion, and the incidence of postoperative blood transfusion was 5.85%. The multivariate regression analysis showed that three or more lumbar fusion segments, preoperative low hemoglobin, with hypertension, lower BMI, and elder people were independent predictors of blood transfusion after PLIF (Table 2). Among them, the AUC of the three or more fusion segments ROC in the training set was 0.745, and the corresponding AUC of the validation set was 0.758. In addition, the AUC of age in the training set was 0.773, while the corresponding AUC in the validation set was 0.778. Age and three or more fusion segments were the main influencing factors of the model, indicating that these two parameters have the greatest impact on the model.

**Table 2:** Univariate and multivariate logistic analyses for risk factors of blood transfusion.

	**Univariable**			**Multivariable**		
	**OR**	**95%CI**	***P***	**OR**	**95%CI**	***p***
**Sex**						
Female	–	–	–			
Male	1.179	0.889–1.564	0.252			
Age	1.094	1.078–1.111	<0.001	1.082	1.065–1.099	<0.001
BMI	0.862	0.825–0.900	<0.001	0.855	0.813–0.899	<0.001
**Comorbidities**						
Hypertension	1.366	1.025–1.820	0.033	1.427	1.028–1.982	0.034
Diabetes mellitus	0.975	0.681–1.397	0.892			
Coronary heart disease	1.410	0.978–2.034	0.065			
Cerebral thrombosis	0.534	0.073–3.927	0.538			
Digestive system diseases	1.449	0.851–2.466	0.172			
Respiratory diseases	0.890	0.534–1.483	0.655			
Other	1.073	0.715–1.609	0.734			
**Indications for surgery**						
Lumbar disc herniation	0.918	0.421–2.004	0.830			
Lumbar spinal stenosis	0.975	0.56–1.699	0.930			
Lumbar spondylolisthesis	1.184	0.665–2.111	0.566			
**Previous history**						
Surgical history	1.628	1.227–2.162	0.001			
Blood transfusion	0.548	0.172–1.746	0.309			
Allergies	0.240	0.033–1.736	0.158			
Smoking	0.960	0.654–1.409	0.836			
Alcohol	0.875	0.565–1.355	0.549			
**ABO**						
A	–	–	–			
AB	1.400	0.858–2.283	0.178			
B	1.148	0.785–1.679	0.475			
O	1.419	0.970–2.074	0.071			
**RH**						
Negative	–	–	–			
Positive	0.340	0.075–1.543	0.162			
**Laboratory tests**						
Hb	0.960	0.953–0.967	<0.001	0.981	0.972–0.989	<0.001
NRBC	0.809	0.703–0.931	0.003			
MCH	1.064	0.991–1.143	0.088			
MCHC	1.001	0.989–1.012	0.902			
MCV	1.037	1.008–1.067	0.012			
WBC	0.919	0.853–0.990	0.027			
PLT	1.000	0.998–1.002	0.910			
**Decompression Fusion Segment**						
1	–	–	–	–	–	–
2	1.565	0.971–2.523	0.066	1.473	0.892–2.433	0.130
≥3	12.889	8.466–19.624	<0.001	12.438	7.893–19.600	<0.001

### Development and Validation of the Nomogram

**Figure 1:**
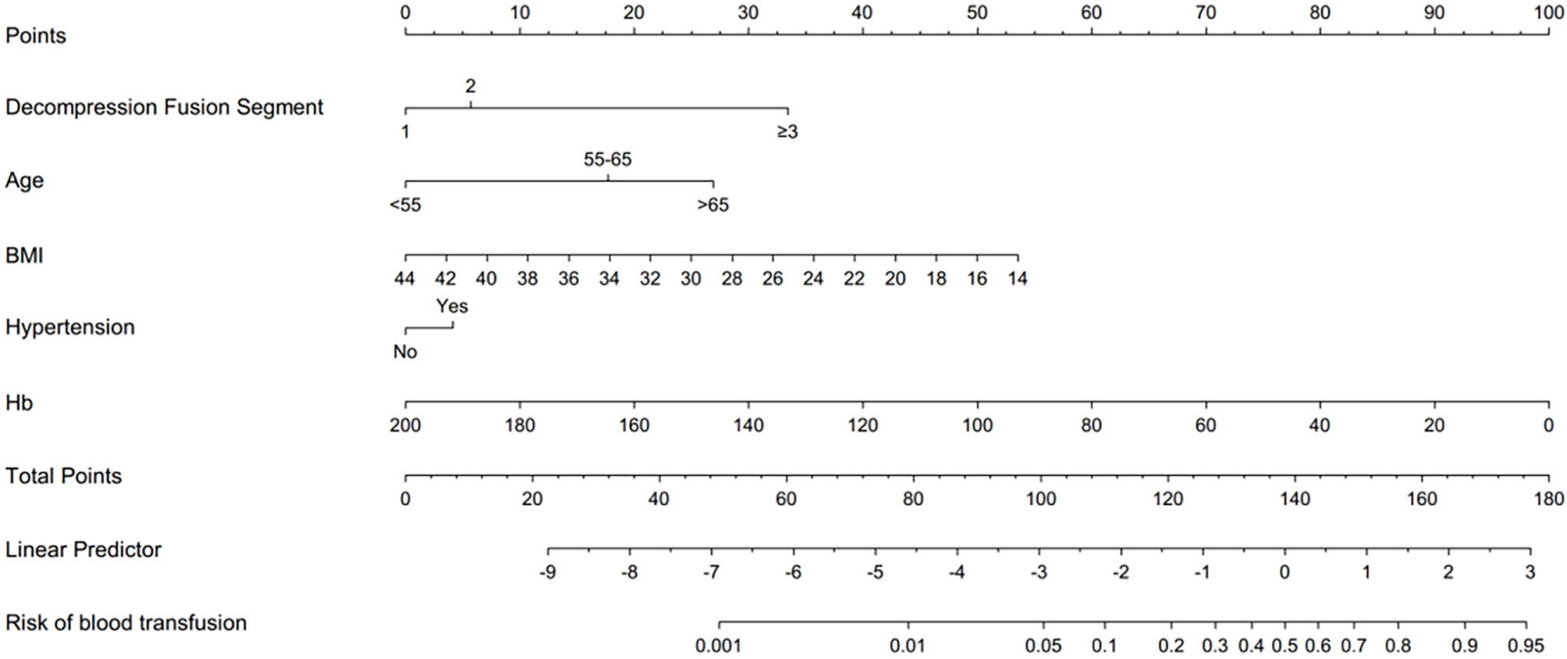
A nomogram based on independent risk factors for predicting transfusion risk.

**Figure 2:**
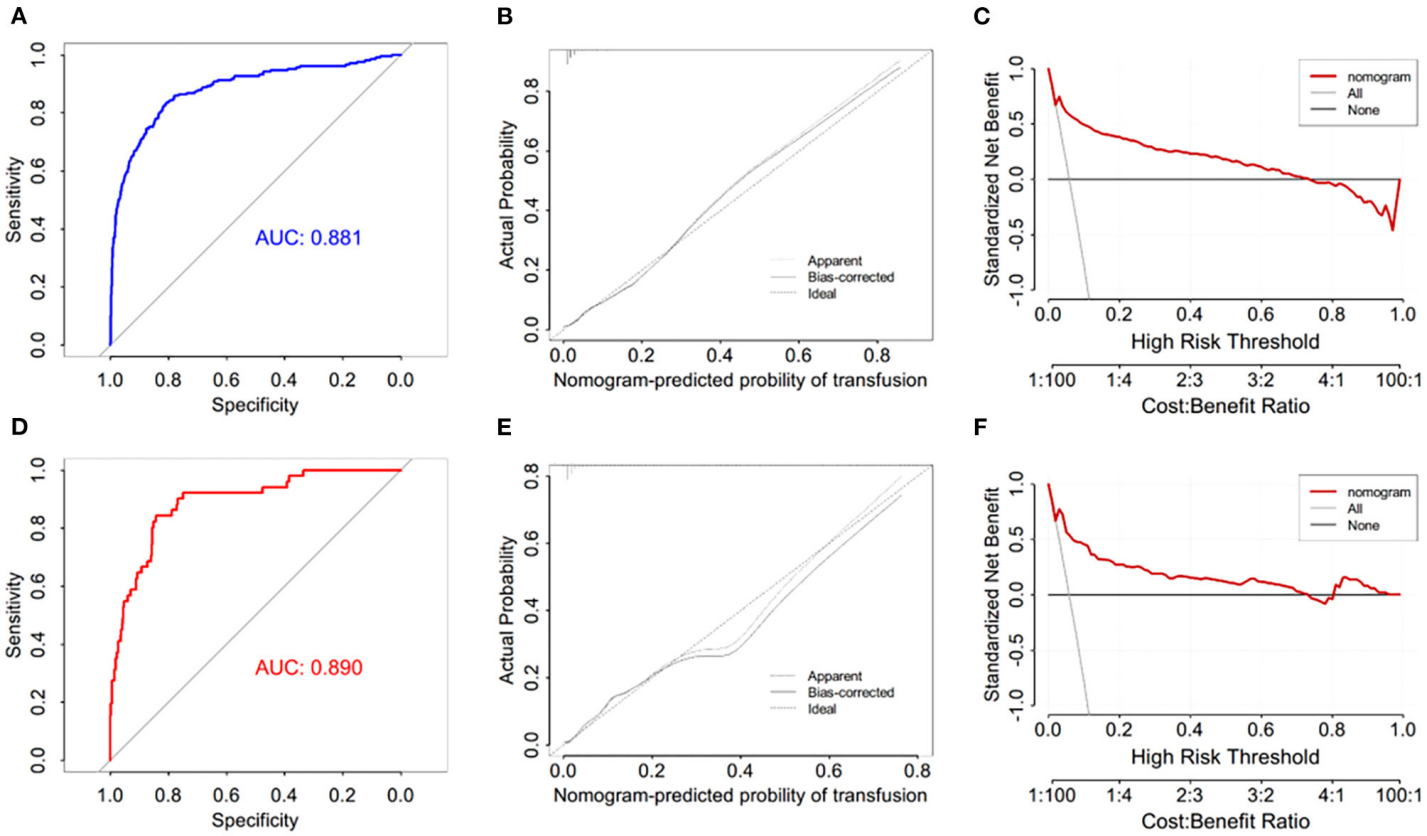
**(A)** ROC curve in training set to evaluate prediction accuracy; **(B)** decision curve analysis in training set; **(C)** calibration curve in training set; **(D)** ROC curve in test set to evaluate prediction accuracy; **(E)** decision curve analysis in test set; **(F)** calibration curve in test set.

We used five independent predictors to build a nomogram (Figure 1). For example, one patient in the clinic, with three or more fusion segments on PLIF, aged over 65 years, BMI is 18 kg/m^2^, hypertension history, and the preoperative hemoglobin was 100 g/L, We calculated the score of each single index, and then, the single item scores could be added together, Total score is 35 + 27.5 + 45 + 5 + 50 = 162.5 points. The probability of perioperative blood transfusion is as high as 85%. In the training set, the AUC of our nomogram was 0.881 (95% CI = 0.853–0.910, *p* < 0.001) (Figure 2A), showing good accuracy in predicting the risk of blood transfusion in patients after lumbar fusion. The calibration curve shows that there is good agreement between the predicted and observed results in terms of the probability of blood transfusion (Figure 2B). Furthermore, DCA shows that there is a net benefit to using this nomogram to predict postoperative blood transfusion if the patient and physician threshold probability is <72% (Figure 2C).

A total of 876 patients were included in the training set, and 51 patients received blood transfusion within 14 days after lumbar fusion. In the training set, the AUC of the nomogram blood transfusion probability prediction model was 0.890 (95% CI = 0.848–0.932, *p* < 0.001) (Figure 2D), and the calibration curve showed that the prediction of blood transfusion probability was in good agreement with the observation (Figure 2E). In addition, the DCA proved that if the threshold probability of patients and doctors is <62%, using a nomogram to predict postoperative blood transfusion has a net benefit (Figure 2F).

### Evaluation the Predictability of Nomogram

**Figure 3:**
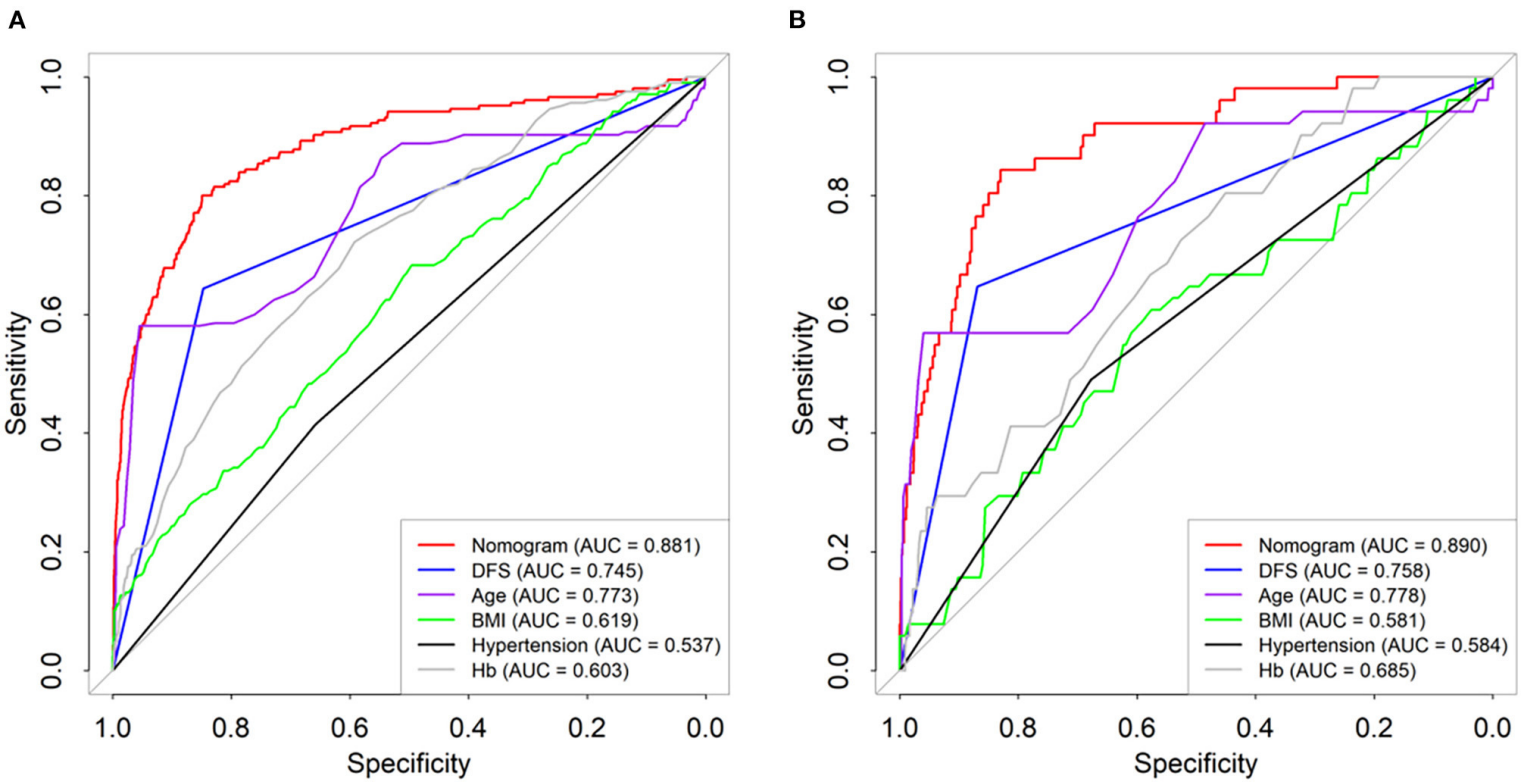
Comparison of the predictive power of different indicators and nomogram plots for transfusion risk in training **(A)** and test datasets **(B)**.

By comparing the ROC curve of the nomogram with the ROCs of other independent risk factors, the results showed that the AUC of the nomogram blood transfusion risk predictor was significantly higher than that of the blood transfusion risk predictor of patients after PLIF (*P* < 0.001) (Figure 3A). Similar to the training set, the AUC was also significantly higher than the AUC of each independent predictor in the validation set (*P* < 0.001) (Figure 3B).

## Discussion

Spine surgery has a long operation time and large associated wounds, especially when dealing with cancellous bone with abundant blood supply, and it is often accompanied by obvious peri-operative bleeding, usually requiring multiple blood transfusions (14). As an important strategy for the treatment of anemia, allogeneic blood transfusion is extremely important to ensure the safety of patients after surgery. The reported incidence of peri-operative blood transfusion is quite different. The incidence in our study was ~3%, which is lower than the 11–18% reported abroad (15, 16) and far lower than the 32.6% reported in China (17). Wong et al. (18) found that the injection of tran-examic acid can effectively reduce the blood loss and blood transfusion rate after PLIF without causing pulmonary embolism and venous thrombosis of the lower extremities.

After reviewing previous relevant literature, we found that Wang et al. (17) concluded that preoperative low hemoglobin, fusion stage, long operation time, and high total intraoperative blood loss are risk factors for blood transfusion. However, the sample size in this model was relatively small, the blood transfusion rate was ~30% higher, and there was no distinction between the training group and the validation group to verify the effectiveness and accuracy of the model. White et al. (19) also found that female sex, age ≥65 years, long-segment fixation and fusion, surgical fixation to the pelvis, and other factors can increase the risk of massive blood loss during surgery. Butler et al. (20) showed that increasing age, ASA grading of multilevel surgery, and prolonged operation time were risk factors affecting lumbar fusion, but neither of them established a predictive model for postoperative blood transfusion. The results of our study show that ≥3 lumbar fusion segments, preoperative low hemoglobin, a history of hypertension, low BMI, and advanced age are risk factors for blood transfusion. This is consistent with domestic and foreign studies on some independent risk factors and establishes and verifies the risk model of blood transfusion after PLIF.

This study found that three or more fusion segments was a risk factor affecting peri-operative blood transfusion. The increase in inter-vertebral fusion segments means an increase in the number of inter-vertebral disc removals. It is often necessary to scrape the endplate cartilage of the upper and lower vertebral bodies of the inserted segments before inserting the inter-vertebral fusion cage. At the same time, it is necessary to extensively strip the paravertebral muscles and soft tissues during the operation to insert the pedicle screws and decompress the spinal canal, which will increase intraoperative bleeding. Morcos et al. (15) also found that multi-segment fusion was an independent risk factor for perioperative blood transfusion, and the blood transfusion rate was 6 times higher. More decompressed and fused segments mean a longer operation time. Many studies have found that the operation time and the number of fusion segments were important predictors of blood transfusion. The probability of blood transfusion increases by 4.2% for every additional hour of operation time. The probability of peri-operative blood transfusion for each additional fusion segment increases by 6 times (16, 21, 22). Aoude et al. (23) showed that the operation time and blood loss were related to an increase in the fusion segments. Therefore, the operation time can be reduced by reducing intra-operative fusion, thereby reducing the risk of peri-operative blood transfusion.

It is worth noting that lower hemoglobin before surgery can also increase the blood transfusion rate of patients during the perioperative period. The lower the red blood cell count, hemoglobin, and hematocrit of the patient before surgery, the poorer their ability to compensate for bleeding during surgery, and the higher the probability that blood transfusions will be required during the peri-operative period. Research by Myers et al. (24) showed that patients with lumbar fusion with preoperative anemia have a higher postoperative infection rate and blood transfusion rate and a longer postoperative hospital stay. In a large-sample retrospective study, Wu et al. (25) showed that preoperative HCT ≤ 39% was associated with an increase in the 30-day postoperative mortality rate. In addition, Carson et al. (26) showed that when Hb ≤ 80 g/L, with every 10 g/L decrease, the risk of death increased by 2.5-fold. Rasouli et al. (27) believed that when preoperative Hb ≤ 100 g/L, the perioperative infection rate increased significantly (~4.23%), the preoperative hemoglobin content was 110–130 g/L, and the postoperative infection rate was significantly reduced (~0.84%). Early identification and correction of preoperative anemia patients is of great significance. We can infuse concentrated red blood cells in advance to supplement hemoglobin to normal levels. Otherwise, patients in the perioperative period are prone to anemia, leading to related complications in multiple organs of the human body, increasing the risk of surgical incision infection, prolonging the hospital stay, and increasing the risk of death.

Older age is one of the risk factors that affect peri-operative blood transfusion. Rasouli et al. (27) showed that age ≥50 years was an independent risk factor for increased blood transfusion risk in the 13,170 patients undergoing lumbar fusion surgery. Yoshihara and Yoneoka (28) showed that middle-aged and elderly patients were more likely to receive allogeneic blood transfusions than middle-aged and young patients. Similarly, Hu et al. (29) collected clinical data from more than 4,000 patients who underwent total knee arthroplasty in the Affiliated Hospital of Qingdao University and found that advanced age was an independent predictor of peri-operative blood transfusion. The older the patient's age is, the worse the hematopoietic function, and the greater the decreases in the lifespan and function of red blood cells. Coupled with the decline of the digestive function of elderly patients, these changes will lead to deficiencies in hematopoietic materials such as iron and vitamins. These findings suggest that once elderly patients undergo lumbar spine surgery, the hematopoietic system cannot replenish blood cells in a short period of time, leading to varying degrees of anemia during the perioperative period. This requires rapid allogeneic blood transfusion treatment.

Patients with lower BMI have relatively low body weight and a relatively low blood volume. The same absolute amount of bleeding leads to an increase in the bleeding score of the patient, and anemia is more likely to occur during the perioperative period. In patients with a low BMI, the proportion of the spine relative to the whole body is relatively large, which may lead to an increase in bleeding scores during spinal surgery (30). The risk of peri-operative blood transfusion in patients with hypertension accompanied by disease is higher than that in patients without hypertension. The author believes that this may be related to changes in the patients' cardiovascular systems caused by hypertension. These patients also have the habit of taking oral anti-hypertensive drugs and anticoagulants such as aspirin. Moreover, these patients are older and have vascular sclerosis, hyalinosis, decreases in capillary contraction and blood clotting abilities, and more intra-operative bleeding (31). Perioperative blood pressure control is not ideal, and the amounts of bleeding and drainage are higher than those in healthy patients.

Our research has some limitations. First, as this is a retrospective study, some data are missing or incomplete, and the conclusions of this study need to be further demonstrated in prospective randomized controlled studies. Second, the sample size of this study was not large enough, and the patient variables were not all included. Furthermore, patients diagnosed with spinal tumors, infection, tuberculosis, trauma fractures, and spinal deformities were excluded from this study. Considering that these groups are more prone to anemia during the perioperative period, it will be necessary to further collect data on patients with these diseases. In addition, postoperative complications such as pneumonia, urinary tract infection, and deep vein thrombosis of the lower extremities were not included. The analysis of the correlations between these complications and blood transfusion is difficult. Our study only included patients from a single medical center; a multicenter study with a large sample size will be required to confirm the results.

## Conclusion

Three or more lumbar fusion segments, preoperative low hemoglobin, with hypertension, lower BMI, and elder people are associated with blood transfusion during the perioperative period. Based on these factors, we established a blood transfusion nomogram and verified that it can be used to assess the risk of blood transfusion after PLIF. It could help clinicians to make clinical decisions and reduce the incidence of perioperative blood transfusion.

## Data Availability Statement

The original contributions presented in the study are included in the article/supplementary material, further inquiries can be directed to the corresponding author/s.

## Author Contributions

BL performed the data analysis. BL and JP wrote the manuscript. BL, HZ, and ZW contributed to the manuscript revise. JP and HZ contributed to literature search and data extraction. BL and ZW conceived and designed the study. All authors contributed to the article and approved the submitted version.

## Conflict of Interest

The authors declare that the research was conducted in the absence of any commercial or financial relationships that could be construed as a potential conflict of interest.

## Publisher's Note

All claims expressed in this article are solely those of the authors and do not necessarily represent those of their affiliated organizations, or those of the publisher, the editors and the reviewers. Any product that may be evaluated in this article, or claim that may be made by its manufacturer, is not guaranteed or endorsed by the publisher.

## Abbreviations

PLIF: Posterior Lumbar Interbody Fusion
DCA: decision curve analysis
ROC: receiver operating characteristic
OR: odds ratio
AUC: area under the curve
CI: confidence interval
BMI: body mass index.
